# A Congenital Anterior Urethrocutaneous Fistula in a Boy Whose Mother Was Exposed to Ionizing Radiations: Case Report and Literature Review

**DOI:** 10.1155/2013/525386

**Published:** 2013-02-26

**Authors:** C. Spinelli, V. Pucci, C. Menchini, I. Buti, L. Fregoli, R. Spisni, A. Mogorovich

**Affiliations:** ^1^Division of Pediatric Surgery, Department of Surgical, Medical, Molecular Pathology and of the Critical Area, 56124 Pisa, Italy; ^2^Department of Urology, University of Pisa, Pisa, Italy

## Abstract

Anterior congenital urethrocutaneous fistula is a rare anomaly that may present in an isolated fashion or in association with other anomalies of the genital urinary tract or anorectal malformations. A case of congenital anterior urethrocutaneous fistula nonassociated with other congenital anomalies in a 3-year-old male whose mother has been exposed to Chernobyl's nuclear fallout is described. The patient was successfully operated with no recurrence. We report a review of the literature about etiology and surgical strategy including the role of ionizing radiations. The congenital anterior urethrocutaneous fistula represents a rare malformation. The etiopathogenesis is unknown.

## 1. Introduction

Primary anterior urethrocutaneous fistula is a very rare malformation with only 28 cases reported in English-language literature from 1962 to 2012 [1–20]. This anomaly is frequently associated with anorectal malformation or other anomalies of the genital-urinary tract, such as hypospadias, chordee, or cryptorchidism. It has been demonstrated that ionizing radiation can be the cause of congenital urogenital malformation. 

We report an additional case of isolated congenital anterior urethrocutaneous fistula discussing its etiology as well as reviewing the literature.

## 2. Case Presentation

**Figure 1:**
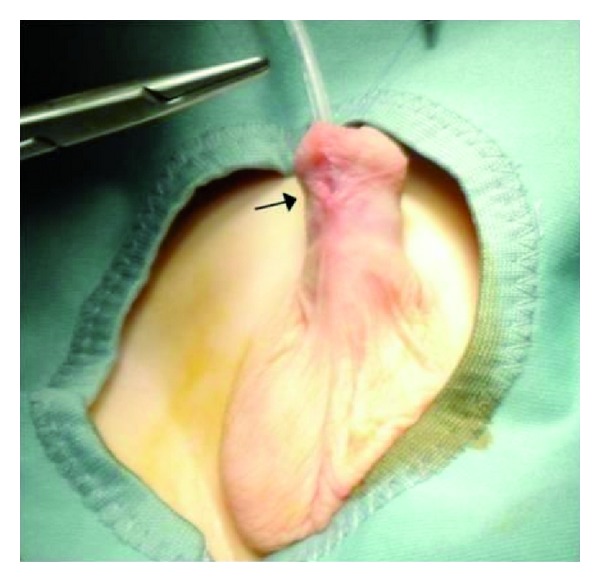
Congenital anterior urethrocutaneous fistula before surgery.

**Figure 2:**
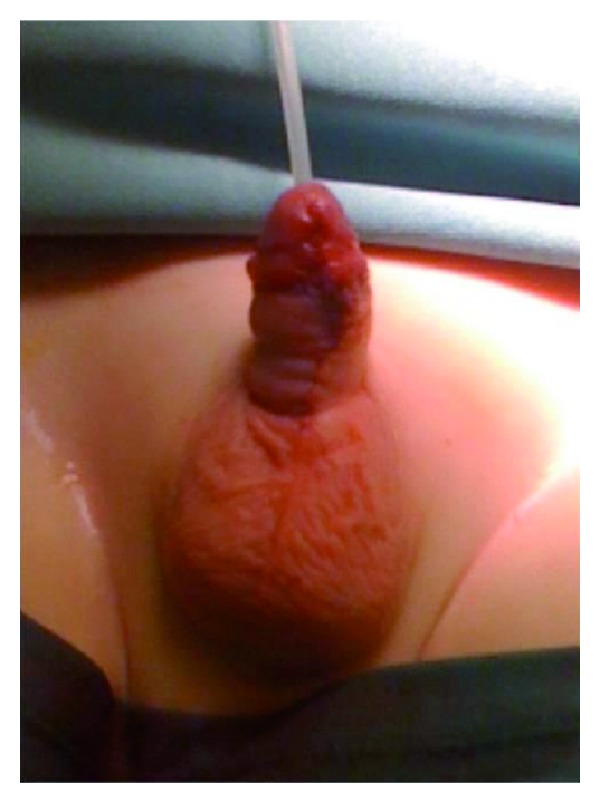
The ventral surface of the penis after immediately surgical correction by one-stage transverse preputial onlay island flap urethroplasty.

A 3 year old, Italian-Ukrainian, not circumcised male came to our attention for the presence of a fistula located on his ventral side of the penis since birth. He had no history of trauma or surgical intervention and his family history did not included hereditary diseases for genital-urinary tract malformations, but the mother was exposed to the nuclear fallout that followed the explosion of the Chernobyl nuclear power plant when she was 1 year old. The systemic and local physical examination of the patient was, except from the fistula, normal; the preputial skin was intact, both testicles were in place and chordee was absent as well as other anomalies. On the ventral side of the midshaft of the penis, an opening measuring 5 × 3 mm was present (Figure 1). It was 2 cm distal to penoscrotal junction and easily passed by a 8 F urethral catheter. The urine was passing mostly through the fistula but also from the external urethral meatus. Investigations such as urine analysis, abdominal ultrasound, and cystourethrography were normal; so, we decided to operate the boy. The fistula was closed by one-stage transverse preputial onlay island flap urethroplasty over the 8 F tube that was kept in place for 7 days (Figure 2). After a 12-month followup, the child is healthy without recurrence.

## 3. Discussion

Anterior urethrocutaneous fistula without anorectal malformation or other anomalies of the genital-urinary tract, such as hypospadias, chordee, or cryptorchidism, is an extremely rare anomaly with just 28 cases previously reported in the literature (Table 1) [1–20].

The etiology of congenital urethrocutaneous fistula is not clear yet as well as the exact development of the male urethra. In male fetuses, sexual differentiation and urethral development approximately begin at the 8th week of development when, under the stimulation of testosterone, the urethral folds begin to close along the midline of ventral surface of the penis. Throughout a similar process, the proximal portion of the granular urethra form shortly thereafter and it is, thus, derived from the urethral plate, endodermal origin. According to the classical theory, the distal portion of the granular urethra is formed by lamellar ingrowth of the surface epithelium, ectodermal origin, which grows toward the distal extent of urethral plate, becoming stratified squamous epithelium around the 15th week. However, this theory for the development of the distal granular urethra has been challenged by the “endodermal differentiation theory.” It has been suggested that the entire penile urethra might differentiate from the fusion of epithelial-mesenchymal interactions [21].

The variability of the patients reported leads to several pathogenetic theories of the congenital urethrocutaneous fistulas development. Campbell [23] proposed that congenital urethrocutaneous fistulas represent embryonal urethral blowouts behind a distal congenital obstruction. Olbourne [2] suggested that focal defect in the urethral plate results in arrested distal migration of the urethral plate or a localized deficiency of a portion of the plate, which prevents fusion of the urethral folds. Goldstein [24] theorized that there is a transient deficiency or inhibition of the local effect of testosterone leading to the failed closure of the urethral groove. Cook and Stephens [25] suggested an alternative mechanism that should be considered, namely, pressure atrophy from the heel of the baby's foot, leading to pressure necrosis. Karnak et al. [5] regarded congenital urethrocutaneous fistulas (excluding those associated with anorectal malformations) as one set of anomalies. Finally, in circumcised patient, possible urethral injuries can be the cause of an acquired urethrocutaneous fistula [25].

Due to the frequent association of congenital urethrocutaneous fistulas with hypospadias and other genitourinary tract anomalies, some genetic pathogenetic theories have been proposed. Genetics studies proved that mutations of ATF 3 (activating transcription factor 3), an estrogens responsive gene expressed during genital development, SHH (sonic Hedge Hog), FGFs 8 and 10 (Fibroblast Growth Factors), Ephrin-B2 and receptors EphB2, Ephb3, often associated with genitourinary anomalies, are also implied in the genesis of isolate urethrocutaneous fistulas [26–29]. Moreover, it has been reported that environmental hazards like ionizing radiation can cause genitourinary tract anomalies, reinforcing the hypothesis concerning the cause-effect relationship between the presence of the congenital anterior urethrocutaneous fistula in our patient and the mother's exposure to ionizing radiation [30, 31].

According to the literature, several surgical techniques for the repair of isolated anterior urethrocutaneous fistula exist including pedicle flap, preputial bound skin flap, modified Denis Browne urethroplasty, or direct closure. The method of repair must be decided on the bases of the location and the size of the fistula and on the presence of other anomalies such as hypospadias or chordee [25]. One of the most important aspects to evaluate is the urethra beyond the fistula. If it is congenitally defective, the simple closure is usually unsuccessful and the fistula is likely to recur. If adequate lateral skin is present, repair can be carried out as one wound, performing a second-stage Johanson's urethroplasty [10]. Otherwise, an onlay flap can be also used. In our case, the fistula was an isolated abnormality and the urethra beyond the fistula was intact; so, a primary closure could have been possible if the dimension of the defect was inferior. This is the reason why we preferred the transverse preputial island flap to repair the lesion; another reason was also because the flap seems to maintain a stable, well-vascularised thick adequately durable tissue to cover the lesion.

The congenital anterior urethrocutaneous fistula represents, as our work and the literature review demonstrate, a rare malformation. The etiopathogenesis is unknown and the case we described is particularly interesting because the mother was exposed, during the pediatric age, to the nuclear fallout that followed the explosion of the Chernobyl nuclear power plant; this opens an interesting and extensive discussion on the possible correlation between the isolated urethrocutaneous fistula and the exposure to ionizing radiations.

## Figures and Tables

**Table 1:** Literature review.

Year	Author	Treatment	Case no.	Recurrence no.
1962	Gupta [1]	Denis Browne	1	—
1976	Olbourne [2]	Denis Browne	2	2
1994	Ritchey et al. [3]	Three-layer closure	1	—
1994	Tannenbaum and Palmer [4]	Thiersch-Duplay	2	—
1995	Karnak et al. [5]	Proximal-based skin flap	1	1
1997	Barwell and Harris [6]	Preputial flap	1	—
1997	Maarafie and Azmy [7]	Preputial flap	1	—
1999	Caldamone et al. [8]	Thiersch-Duplay	6	—
2000	Nakane et al. [11]	Preputial flap	1	—
2000	Sharma et al. [9]	Two-layer closure	1	—
2000	Harjai [10]	Preputial flap	1	—
2003	Betalli et al. [12]	Three-layer closure	1	1
2004	Agarwal [13]	Local skin turndown flap	1	—
2005	Akman et al. [14]	Preputial flap	1	—
2006	Ceylan et al. [15]	Preputial flap	1	—
2008	Rashid et al. [16]	Thiersch-Duplay	1	—
2008	Arena et al. [17]	Three-layer closure	1	—
2013	Spinelli et al. [22]	Preputial flap	1	—
2009	Galinier et al. [18]	Bladder mucosal graft	1	—
2011	Jindal et al. [19]	Two-layer closure	1	—
2011	Chen et al. [20]	Local skin turndown flap	1	—

Total			28	4
